# Comparison of prognosis after transurethral resection of bladder tumor between solitary and multiple bladder cancers

**DOI:** 10.1097/MD.0000000000040314

**Published:** 2024-11-01

**Authors:** Mingxin Diao, Yue Li, Zihui Gao, Chunji Wang, Yaming Gu

**Affiliations:** a Department of Urology, Peking University First Hospital-Miyun Hospital, Beijing, China; b Department of Urology, The Third Hospital of Hebei Medical University, Shijiazhuang, China.

**Keywords:** multiple bladder cancer, prognosis, solitary bladder cancer, transurethral resection of bladder tumor

## Abstract

This study investigates the difference in overall survival rates after transurethral resection of bladder tumor between solitary and multiple bladder cancers, aiming to provide guidance for clinical practitioners. A retrospective analysis was conducted on 133 patients with bladder cancer who underwent transurethral resection of bladder tumor from April 2017 to October 2023, of which 112 patients had complete clinical and follow-up data. Clinical and follow-up data were collected, and the overall survival rates after surgery were compared between solitary and multiple bladder cancers. In this study, the recurrence rate after transurethral resection of bladder tumor was 23.21% (26/112), and the overall survival rate was 80.36% (90/112). The overall survival rate after transurethral resection of bladder tumor was 92.11% (35/38) in the solitary bladder cancer group and 74.32% (55/74) in the multiple bladder cancer group, with a statistically significant difference between the 2 groups (*P* = .025). The proportion of high-grade pathology was 7.89% (3/38) in the solitary bladder cancer group and 25.68% (19/74) in the multiple bladder cancer group after transurethral resection of bladder tumor, with a statistically significant difference between the 2 groups (*P* = .025). The mean tumor diameter after transurethral resection of bladder tumor was 2.76 ± 1.66 cm in the solitary bladder cancer group and 4.04 ± 3.17 cm in the multiple bladder cancer group, with a statistically significant difference between the 2 groups (*P* = .023). Univariate and multivariate regression analyses revealed that the number of bladder tumors is a risk factor for overall survival after bladder cancer surgery (*P* = .004). Multiple bladder cancers have a higher pathological grade, larger tumor diameter, and poorer prognosis after transurethral resection of bladder tumor compared to solitary bladder cancers. The number of bladder tumors is an independent risk factor for overall survival after bladder cancer surgery.

## 1. Introduction

Bladder cancer is the 10th most common cancer and the 13th most common cause of cancer deaths, and because of its high rate of recurrence, it imposes a significant financial and health burden on patients.^[1,2]^ Due to the inherently aggressive nature of bladder cancer and its susceptibility to recurrence, patients usually require multimodality and invasive treatments, such as repeated transurethral cystectomy of bladder tumors combined with bladder perfusion chemotherapy, but the 5-year recurrence-free survival rate is still <43%.^[3–5]^ Bladder cancer is classified into non-muscle-invasive bladder cancer and muscle-invasive bladder cancer based on the depth of infiltration into the bladder tissue, with the latter exhibiting a higher tendency for distant metastasis and poorer prognosis.^[6–8]^

Currently, the distinction of bladder cancer primarily focuses on non-muscle-invasive and muscle-invasive categories, with limited attention given to the differentiation between solitary and multiple bladder cancers. However, the number of bladder lesions serves as a crucial determinant of bladder cancer severity, significantly impacting treatment selection, and postoperative outcomes.^[9]^

This study investigates the impact of tumor number in bladder cancer on postoperative patient survival and analyzes the risk factors for survival after transurethral resection of bladder tumors, providing references for clinical treatment.

## 2. Methods

A total of 133 patients with bladder cancer underwent transurethral resection of bladder tumor at the Department of Urology, Peking University First Hospital-Miyun Hospital, from April 2017 to October 2023, among which 112 patients had complete baseline and follow-up data. Among the bladder cancer patients also 38 patients with solitary bladder cancer and 74 patients with multiple bladder cancer. Multiple bladder cancers are defined as masses greater than or equal to 2 in the bladder. Clinical and follow-up data were collected, including gender, age, body mass index, smoking status, hypertension, diabetes, pathological staging, pathological grading, and tumor diameter. Pathological staging and grading for multiple bladder cancer were based on the most malignant lesion. Postoperative follow-up included assessment of recurrence and survival.

Inclusion criteria: 1. Pathological diagnosis of bladder cancer; 2. Complete clinical and follow-up data.

Exclusion criteria: 1. Patients with a history of other tumors; 2. Abnormal coagulation function; 3. Cardiopulmonary dysfunction.

This study was conducted in accordance with the principles of the Helsinki Declaration (revised in 2013) and was approved by the Ethics Committee of Peking University First Hospital-Miyun Hospital. Informed consent for this retrospective analysis was waived.

### 2.1. Surgical technique

After successful anesthesia, lithotomy position was taken, and routine disinfection was performed. A F24 12° resectoscope (Hawk, Hangzhou, China) was inserted into the bladder via the urethra, and the bladder was filled and irrigated with mannitol to examine its condition and locate the tumors. Tumors were resected with the resectoscope, with a margin of 1 cm beyond the tumor border and reaching the superficial muscle layer. Tissue specimens were flushed out, and hemostasis was achieved by thorough electrocoagulation to check for bleeding and residual tissue. The resectoscope was then removed, and a 3-way Foley catheter (Bard, NJ) was left in place for bladder irrigation.

### 2.2. Follow-up

Patients with minimal postoperative bladder bleeding underwent bladder irrigation for 1 day, while those with significant bleeding underwent irrigation for 2 days. Follow-up was conducted via telephone to assess postoperative tumor recurrence and survival.

### 2.3. Statistical analysis

Statistical analysis was performed using SPSS version 22.0 (IBM Corp., Armonk, NY). Quantitative variables, including age, body mass index, and tumor diameter, were expressed as mean ± standard deviation for normally distributed data and as median for skewed data. For continuous variables, *t* tests were used for normally distributed variables, while the Mann–Whitney *U* test was used for non-normally distributed variables. Fisher exact probability test was used for categorical variables.

Univariate and multivariate regression analyses were used to identify independent risk factors for overall survival in bladder cancer patients following transurethral resection of bladder tumors. A *P*-value < .05 was considered statistically significant.

## 3. Results

### 3.1. Baseline characteristics

Baseline characteristics of the patients are presented in Table 1. A total of 112 patients with bladder cancer were included in this study, of which 38/112 (33.93%) had solitary bladder cancer and 74/112 (66.07%) had multiple bladder cancer. The recurrence rate after transurethral resection of bladder tumor was 23.21% (26/112), and the overall survival rate was 80.36% (90/112).

**Table 1:** Basic characteristics of the patients.

Variable	Mean (SD) or n/N
Patients	112
Mean age (years)	69.32 ± 9.52
BMI (kg/m^2^)	24.65 ± 3.06
Sex, n (%)	
Male	93 (83.04％)
Female	19 (16.96％)
Hypertension, n (%)	
Yes	46 (41.07％)
No	66 (58.93％)
Diabetes mellitus, n (%)	
Yes	11 (9.82％)
No	101 (90.18％)
Smoking, n (%)	
Yes	33 (29.46％)
No	79 (70.54％)
Number of tumors, n (%)	
Single	38 (33.93％)
Multiple	74 (66.07％)
Pathological T, n (%)	
T1–T2	90 (80.36％)
T3–T4	22 (19.64％)
Histological grade, n (%)	
G1–G2	38 (33.93％)
G3–G4	74 (66.07％)
Tumor diameter (cm)	3.61 ± 2.83
Postoperative recurrence, n (%)	
Yes	26 (23.21％)
No	86 (76.79％)
Postoperative death, n (%)	
Yes	22 (19.64％)
No	90 (80.36％)

BMI = body mass index.

### 3.2. Comparison of overall survival rates after transurethral resection of bladder tumor between solitary and multiple bladder cancer groups

Clinical data of patients in the solitary and multiple bladder cancer groups are presented in Table 2. The overall survival rate after transurethral resection of bladder tumor was 92.11% (35/38) in the solitary bladder cancer group and 74.32% (55/74) in the multiple bladder cancer group, with a statistically significant difference between the 2 groups (*P* = .025). The proportion of high-grade pathology was 7.89% (3/38) in the solitary bladder cancer group and 25.68% (19/74) in the multiple bladder cancer group, with a statistically significant difference between the 2 groups (*P* = .025). The mean tumor diameter after transurethral resection of bladder tumor was 2.76 ± 1.66 cm in the solitary bladder cancer group and 4.04 ± 3.17 cm in the multiple bladder cancer group, with a statistically significant difference between the 2 groups (*P* = .023). The recurrence rate of overall survival after transurethral resection of bladder tumor was 23.68% (9/38) in the solitary bladder cancer group and 22.97% (17/74) in the multiple bladder cancer group, with no statistically significant difference between the 2 groups (*P* = .875). Univariate and multivariate regression analyses revealed that the number of bladder tumors is an independent risk factor for overall survival after bladder cancer surgery (*P* = .004, Table 3).

**Table 2:** Comparing the difference in prognosis between the single and the multiple bladder cancer group.

Variable	Solitary bladder cancer group	Multiple bladder cancer group	*P* value
Patients	38 (33.93％)	74 (66.07％)	
Mean age (years)	71.08 ± 8.71	68.42 ± 9.74	.166
BMI (kg/m^2^)	24.73 ± 3.82	24.61 ± 2.55	.198
Sex, n (%)			.768
Male	31 (81.58％)	62 (83.78％)	
Female	7 (18.42％)	12 (16.22％)	
Hypertension, n (%)			.29
Yes	13 (34.21％)	33 (44.59％)	
No	25 (65.73％)	41 (55.41％)	
Diabetes mellitus, n (%)			.395
Yes	5 (13.16％)	6 (8.11％)	
No	33 (86.84％)	68 (91.89％)	
Smoking, n (%)			.22
Yes	14 (38.84％)	19 (25.68％)	
No	24 (63.16％)	55 (74.32％)	
Pathological T, n (%)			.025
T1–T2	35 (92.11％)	55 (74.32％)	
T3–T4	3 (7.89％)	19 (25.68％)	
Histological grade, n (%)			.641
G1–G2	14 (36.84％)	24 (32.43％)	
G3–G4	24 (63.16％)	50 (67.57％)	
Tumor diameter (cm)	2.76 ± 1.66	4.04 ± 3.17	.023
Postoperative recurrence, n (%)			.875
Yes	9 (23.68％)	17 (22.97％)	
No	29 (76.32％)	57 (77.03％)	
Postoperative death, n (%)			.025
Yes	3 (7.89％)	19 (25.68％)	
No	35 (92.11％)	55 (74.32％)	

BMI = body mass index.

**Table 3:** Univariate analysis and multivariate analysis of overall survival of bladder cancer.

Characteristic	Univariate analysis		Multivariate analysis	
	HR (95% CI)	*P*-value	HR (95% CI)	*P*-value
Smoking, yes vs no	4.992 (1.091–22.834)	.038	4.500 (0.891–22.732)	.069
Hypertension, yes vs no	2.000 (0.711–5.625)	.189		
Diabetes, yes vs no	0.585 (0.141–2.426)	.460		
Tumor diameter	0.981 (0.826–1.166)	.830		
Age	1.603 (0.608–4.231)	.340		
BMI	0.920 (0.787–1.076)	.298		
Tumor number,≥ 2 vs <2	1.103 (1.035–1.175)	.003	1.112 (1.035–1.194)	.004
Sex, male vs female	0.839 (0.247–2.847)	.778		
Pathological T, T3 + T4 vs T1 + T2	0.789 (0.255–2.440)	.681		
Pathological N, N1–3 vs N0	0.109 (0.024–0.502)	.004	0.100 (0.019–0.532)	.007
Histological grade, high vs low	0.968 (0.354–2.654)	.949		

## 4. Discussion

Bladder cancer is one of the most common malignant tumors of the urinary system, and its incidence is on the rise worldwide.^[10–12]^ Despite various treatment modalities available, including surgical resection, chemotherapy, radiotherapy, and immunotherapy, some patients still face the risks of recurrence, metastasis, and poor prognosis.^[13–18]^ This study aims to explore the potential factors contributing to the poorer prognosis of multiple bladder cancer by comparing the prognosis after transurethral resection between solitary and multiple bladder cancers. A deeper understanding of the association between multiple tumors and bladder cancer will assist clinicians in better diagnosing and managing this complex disease entity, thereby providing more personalized and effective treatment strategies for patients.

In this study, a comparison between solitary and multiple bladder cancers revealed a lower overall survival rate in multiple bladder cancer (55/74, 74.32%) compared to solitary bladder cancer (35/38, 92.11%), with a statistically significant difference between the 2 groups (*P* = .025). Similar findings were reported by F Millán-Rodríguez et al^[19]^ and Stefan Denzinger et al,^[20]^ who identified multiple bladder cancer as a prognostic risk factor. Roberto Contieri et al^[21]^conducted a multivariate regression analysis and discovered that multifocal bladder tumors are independently associated with disease progression. This may be attributed to the higher pathological staging associated with multiple bladder cancers, which typically reflects a higher degree of biological malignancy of the tumors, including enhanced proliferative capacity and increased ability to infiltrate lymphatic vessels or blood vessels.^[22]^ Tumors with higher pathological staging often accompany reinforced immune escape mechanisms, making them more prone to evading the host’s immune attack. Consistently, our study also found a higher proportion of high-grade pathology in multiple bladder cancer patients compared to solitary bladder cancer patients (*P* = .025), indicating a potential association between the poorer prognosis of multiple bladder cancer and its higher pathological staging.

However, no significant correlation was found between multiple bladder cancer and postoperative recurrence in our study (*P* = .876). Oscar Rodríguez Faba et al^[23]^ found that both multiple bladder cancer and tumor diameter >3 cm were risk factors for postoperative recurrence. This may be related to the quality of transurethral resection surgery, as demonstrated in the study by Brausi et al,^[24]^ which showed significant variation in postoperative recurrence rates among different hospitals.

Smoking is an independent risk factor for the development of bladder cancer, but in our study, smoking was not found to directly influence the number of bladder tumors.^[2,25,26]^ Poletajew et al^[27]^ analyzed 2160 patients who underwent transurethral resection of bladder tumors and found age and gender as risk factors for postoperative survival but did not find an association with the number of bladder tumors, consistent with our study results.

The high pathological staging and poorer prognosis of multiple bladder cancer warrant attention. For multiple tumors, the importance of postoperative follow-up examinations should be emphasized, including both cystoscopy and tumor marker testing.

This study has certain limitations. Firstly, the sample size was relatively small, which may introduce some bias in the analysis. Additionally, as the study was conducted at a single center without multicenter data, the generalizability of the study findings may be limited.

## 5. Conclusions

Multiple bladder cancer exhibits higher pathological staging, larger tumor diameter, and poorer prognosis after transurethral resection compared to solitary bladder cancer. The number of bladder tumors is an independent risk factor for overall survival after bladder cancer surgery.

## Author contributions

**Conceptualization:** Mingxin Diao, Yue Li, Zihui Gao.

**Data curation:** Yaming Gu, Mingxin Diao, Yue Li, Zihui Gao.

**Formal analysis:** Yue Li.

**Investigation:** Yaming Gu, Mingxin Diao, Zihui Gao.

**Methodology:** Yaming Gu, Mingxin Diao, Chunji Wang.

**Project administration:** Yue Li, Chunji Wang.

**Software:** Yaming Gu, Mingxin Diao.

**Validation:** Mingxin Diao.

**Visualization:** Mingxin Diao.

**Writing – original draft:** Mingxin Diao.

**Writing – review & editing:** Yaming Gu, Mingxin Diao.
